# The Dark Side of UV-Induced DNA Lesion Repair

**DOI:** 10.3390/genes11121450

**Published:** 2020-12-02

**Authors:** Wojciech Strzałka, Piotr Zgłobicki, Ewa Kowalska, Aneta Bażant, Dariusz Dziga, Agnieszka Katarzyna Banaś

**Affiliations:** 1 Department of Plant Biotechnology, Faculty of Biochemistry, Biophysics and Biotechnology, Jagiellonian University, Gronostajowa 7, 30-387 Krakow, Poland; wojciech.strzalka@uj.edu.pl (W.S.); piotr.zglobicki@uj.edu.pl (P.Z.); ewa.b.kowalska@uj.edu.pl (E.K.); aneta.bazant@doctoral.uj.edu.pl (A.B.); 2 Department of Microbiology, Faculty of Biochemistry, Biophysics and Biotechnology, Jagiellonian University, Gronostajowa 7, 30-387 Krakow, Poland; dariusz.dziga@uj.edu.pl

**Keywords:** DNA damage, dark DNA repair, BER, NER, MMR, NHEJ, HR, TLS, UV

## Abstract

In their life cycle, plants are exposed to various unfavorable environmental factors including ultraviolet (UV) radiation emitted by the Sun. UV-A and UV-B, which are partially absorbed by the ozone layer, reach the surface of the Earth causing harmful effects among the others on plant genetic material. The energy of UV light is sufficient to induce mutations in DNA. Some examples of DNA damage induced by UV are pyrimidine dimers, oxidized nucleotides as well as single and double-strand breaks. When exposed to light, plants can repair major UV-induced DNA lesions, i.e., pyrimidine dimers using photoreactivation. However, this highly efficient light-dependent DNA repair system is ineffective in dim light or at night. Moreover, it is helpless when it comes to the repair of DNA lesions other than pyrimidine dimers. In this review, we have focused on how plants cope with deleterious DNA damage that cannot be repaired by photoreactivation. The current understanding of light-independent mechanisms, classified as dark DNA repair, indispensable for the maintenance of plant genetic material integrity has been presented.

## 1. Introduction

**Figure 1:**
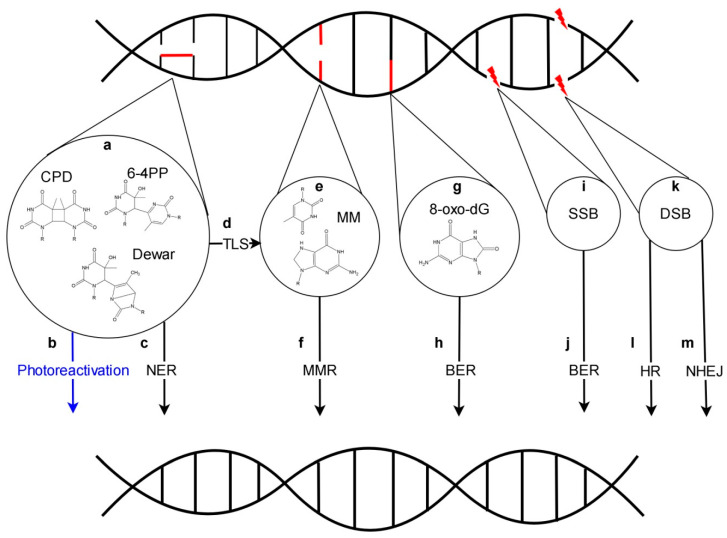
A scheme of DNA damage directly and indirectly caused by UV and network of mechanisms involved in their repair. UV irradiation causes formation of pyrimidine dimers (**a**) that can be repaired either by photoreactivation under UV-A/blue light (**b**) or NER (nucleotide excision repair) (**c**). Alternatively, pyrimidine dimers can be bypassed during replication by TLS (translesion synthesis) (**d**), which leads to production of mismatched bases (**e**). Mismatches are repaired by MMR (mismatch repair) (**f**). UV-induced oxidative stress may lead to formation of 8-oxo-dG (**g**) that are repaired via BER (base excision repair) (**h**). UV may also indirectly cause formation of single strand breaks (**i**) repaired by proteins involved in BER pathway (**j**) and double strand breaks (**k**), that may be repaired by HR (homologous recombination) (**l**) or NHEJ (non-homologous end joining) (**m**). Black arrows represent the mechanisms of dark repair, while photoreactivation is marked in blue.

Solar light is indispensable for life on Earth. The blue and red light absorbed by photosynthetic machinery serves as a source of energy used for biomass production. However, beside the visible range including photosynthetically active radiation, the Sun also emits ultraviolet radiation which is harmful to the living organisms. Three types of UV (ultraviolet) light may be distinguished based on their biological activity: UV-A spectrum (from 315 to 400 nm), UV-B (from 280–315 nm) and UV-C (from 100 to 280 nm) [1,2,3]. The surface of the Earth is protected against UV irradiation by the ozone layer which is present in the upper part of the atmosphere. The ozone layer absorbs the whole UV-C radiation, most of UV-B and a small fraction of UV-A. After solar light goes through the ozone layer, around 5.7% and 0.3% of sunlight energy is in UV-A and UV-B range, respectively, when measured at the sea level [4]. The exposure of living organisms to UV radiation leads to the formation of different types of DNA lesions (Figure 1). Most of them are pyrimidine dimers of which CPDs (cyclobutane pyrimidine dimers) are the most common, followed by 6-4 PPs (pyrimidine (6-4) pyrimidone photoproducts) which can isomerize into Dewar photoproducts [5,6,7,8]. In addition, 8-oxo-dG (8-oxo-7,8-dihydro-2′-deoxyguanosine), FapyAde (e.g., 4,6-diamino-5-formamidopyrimidine), uracil, SSBs (single-strand breaks) and DSBs (double-strand breaks) are formed [5,9,10,11]. The presence of 8-oxo-dG in the DNA template results mainly in G-C to T-A transversion during DNA replication [12]. Moreover, when located in proximity to other DNA lesions, it may inhibit their repair leading to serious defects in cell functioning [13].

Among the mechanisms used by plants to counteract the negative effects of UV exposure are DNA repair systems. The fact that plant cells can effectively repair selected types of UV-induced DNA lesions via photoreactivation has been well documented. According to our knowledge, the activity of this repair system in plants is limited to the repair of CPDs, 6-4 PPs and Dewar photoproducts only [14,15,16]. Photoreactivation is performed by photolyases which use UV-A/blue light energy to simply reverse pyrimidine dimers formed under UV. These enzymes have been found in most prokaryotes and eukaryotes except placental mammals. Despite the proven role of UV-A/blue light dependent photoreactivation in the maintenance of plant genome integrity, it should be emphasized that this process is not always sufficient to cope with all the deleterious events caused by UV in DNA [6]. First of all, the effectiveness of the photoreactivation system is dependent on changing environmental conditions which modulate the amount of solar light reaching plant cells. Photoreactivation does not work in extreme deficiency of light. Moreover, to cope with DNA lesions formed indirectly by UV irradiation, e.g., 8-oxo-dG, SSBs or DSBs which are not repaired by photolyases, plants have to activate other rescue mechanisms. Over the past decades, numerous eukaryotic proteins engaged in light-independent neutralization (also referred to as dark repair) of DNA lesions caused by UV have been identified (reviewed: [17]). These proteins belong to different pathways which are responsible for the maintenance of genomic stability including NER (nucleotide excision repair), BER (base excision repair), MMR (mismatch repair), NHEJ (non-homologous end joining), HR (homologous recombination) and TLS (translesion synthesis). Additional difficulty in understanding how plants repair various types of damage in DNA arises from differences in the expression of genes involved in DNA repair between plant organs and life stages. It was demonstrated that genes responsible for light-dependent DNA repair, encoding CPD and 6-4 PP specific photolyases, are expressed both in young and mature leaves [18,19]. The genes coding NER and BER proteins involved in dark repair are expressed more strongly in the proliferating tissues of SAM (shoot apical meristem) than in mature leaves. On the other hand, the transcript levels of some genes of MMR pathway were found to be higher in mature leaves compared to SAM [18]. In this review, we have presented the current level of cognition on the mechanisms responsible for dark repair of UV-induced DNA lesions in plants.

## 2. Dark DNA Repair

Repair of UV-induced DNA lesions is performed by a network of specialized proteins responsible, among others, for the recognition of errors, excision of incorrect sequences, synthesis of missing DNA fragments using an undamaged strand as a template and its ligation with the DNA backbone. Under light conditions, pyrimidine dimers are repaired mainly by photoreactivation, while the prominent role of NER becomes apparent in the dark. BER operates mainly on SSBs and oxidized derivatives including 8-oxo-dG. DSBs are corrected by NHEJ and HR. Finally, bypass of pyrimidine dimers by TLS pols (polymerases) may produce mismatches, which in turn are substrates for MMR (Figure 1).

## 3. Nucleotide Excision Repair

**Figure 2:**
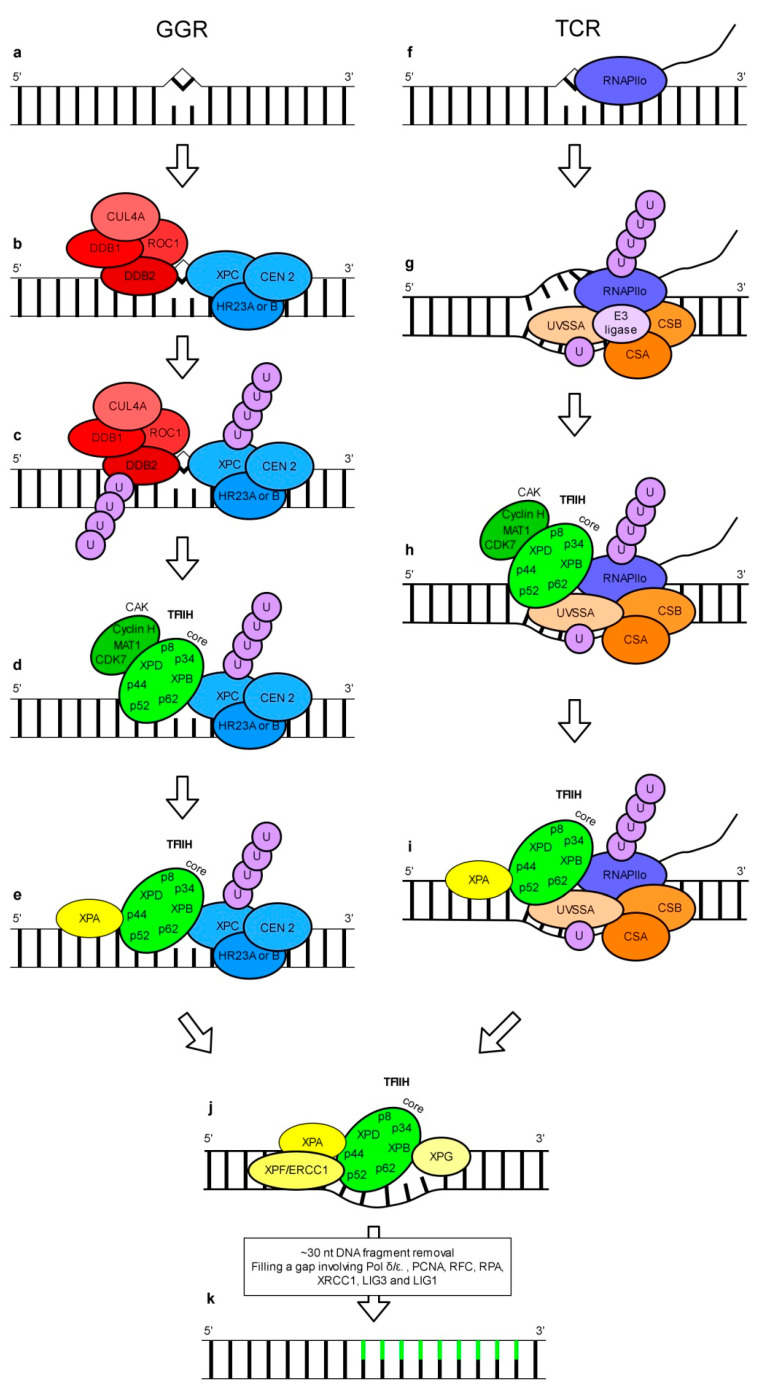
A simplified mechanism of the GGR (global genome repair) and TCR (transcription-coupled repair) subpathways of NER based on a mammalian model. Detection and processing of a DNA lesion in GGR (**a**–**e**). Bulky DNA damage (e.g., 6-4 PP) changes the DNA structure (**a**). Such a lesion is recognized by the XPC-HR23-CEN2 (*Xeroderma pigmentosum*, complementation group C-UV excision repair protein RAD23- centrin 2) complex. The DDB1-DDB2-CUL4-ROC1 (damaged DNA-binding-cullin 4A-homeobox-leucine zipper protein ROC1) complex assists in the recognition of other lesions such as CPDs (cyclobutane pyrimidine dimers) (**b**). DDB2 and XPC are polyubiquitinated by E3 ligase activity of the UV-DDB complex composed of DDB1 and DDB2 proteins (**c**). The TFIIH (transcription factor II H) complex consisting of a kinase subcomplex—CAK (CDK (cyclin-dependent kinase)-activating kinase) and a core complex is recruited to the site of the lesion while the DDB1-DDB2-CUL4-ROC1 complex dissociates (**d**). Upon the recruitment of XPA, CAK is released (**e**). Detection and processing of a DNA lesion in TCR (**f**–**i**). During transcription, RNAPII (RNA polymerase II) movement is blocked by a DNA lesion (**f**). Stalling of RNAPII (RNAPIIo) results in the recruitment of CSA (Cockayne syndrome, Group A), CSB, UVSSA (UV-stimulated scaffold protein A) and cullin-ring type E3 ligase. The CSA and CSB complex facilitates cullin-ring type E3 ligase-mediated ubiquitination of UVSSA and RPB1 (RNA polymerase II subunit B1), a subunit of RNAPIIo (**g**). TFIIH is recruited to the site of the lesion and associates with RNAPIIo (**h**). This is followed by the recruitment of XPA and release of CAK (**i**), compare (**e**). The final NER steps are common for both pathways: simultaneously with the recruitment of XPG and XPF-ERCC1 (excision repair cross-complementation group 1) to TFIIH a release of the XPC-HR23-CEN2 (GGR) or CSA-CSB-UVSSA and RNAPIIo (TCR) occurs. The 3′ and 5′ sides of the DNA damage are incised by the XPG and XPF-ERCC1 complex, respectively (**j**). An about 30-nt-long fragment of DNA is removed. Gap filling requires the involvement of a wide repertoire of proteins involved in DNA metabolism including pol *δ* or pol *ε*, PCNA (proliferating cell nuclear antigen), DNA LIG1 (DNA ligase 1), RFC (replication factor C), RPA (replication protein A), XRCC1 (X-ray repair cross-complementing protein 1) and DNA LIG3 (**k**).

This DNA repair pathway can recognize and repair a wide range of structurally unrelated lesions including CPDs and 6-4 PPs [20]. The concept of NER involvement in the repair of UV-induced DNA damage is based mainly on studies using human, animal and yeast models. Two subpathways of NER called GGR (global genome repair) and TCR (transcription-coupled repair) have been discovered (Figure 2). GGR and TCR operate on the entire genome and transcriptionally active regions of the genome, respectively.

In the human GGR subpathway DNA lesions are recognized by a protein complex called XPC-HR23-CEN2 which is composed of XPC (*Xeroderma pigmentosum*, complementation group C), CEN2 (centrin 2) and either HR23A or HR23B (UV excision repair protein RAD23 homolog A or B) (Figure 2b) [21,22]. This complex can detect different bulky DNA lesions including 6-4 PPs [23,24], most probably by recognizing specific secondary DNA structures [25]. This assumption explains the wide spectra of unrelated substrates recognized by the XPC-HR23-CEN2 complex. Surprisingly, detailed studies have revealed that CPDs, which are the most frequently UV-induced DNA lesions, are inefficiently bound by this XPC-HR23-CEN2 complex [24]. This problem may be solved with the help of the UV-DDB (UV-damaged DNA-binding) complex. UV-DDB is a heterodimer made up of DDB1 and DDB2 proteins [26,27]. It binds various types of DNA damage, including CPDs and 6-4 PPs [28,29,30,31,32]. Interestingly, UV-DDB can bind DNA lesions localized both in tightly packed nucleosomes and in linker regions but with different binding affinity [33]. Ectopic expression of cDNA coding for human DDB2 in hamster ovary cells, whose own DDB2 protein is inactive, enhances the removal of CPDs from genomic DNA [34,35]. In cells with a mutation causing defected binding of UV-DDB to DNA, the repair of 6-4 PPs via GGR was only moderately impaired while the removal of CPDs was significantly reduced [36]. DDB2 is thought to be necessary for the repair of CPDs in vivo due to the recruitment of XPC [37,38]. However, the role of UV-DDB in the repair of pyrimidine dimers by GGR is unequivocal. As shown in experiments using a cell-free system, while CPD repair via NER does not require the UV-DDB complex [39], the repair of 6-4 PPs is actually inhibited by this complex [31]. Other results showed that in vitro the excision of CPDs and 6-4 PPs was stimulated by UV-DDB [40,41]. These results reveal a more complex role of UV-DDB than simply in assisting the recognition of DNA lesions by XPC-HR23-CEN2 complex. The UV-DDB heterodimer is a part of a larger complex which also includes CUL4 (cullin 4A) and ROC1 (homeobox-leucine zipper protein ROC1) (Figure 2b). The UV-DDB-CUL4-ROC1 complex displays ubiquitin E3 ligase activity which is regulated by CNS (COP9 signalosome) associated with this complex [42]. Studies of Sugasawa and co-workers [31] using a human cell-free system, showed UV-DDB-CUL4-ROC1-dependent polyubiquitination of UV-DDB and XPC in vitro (Figure 2c). This polyubiquitination weakens and enhances the affinity of UV-DDB and XPC, respectively, to 6-4 PPs. The UV-DDB-CUL4-ROC1 complex is also responsible for histone modification. Histone ubiquitination leads to changes in chromatin stability and is important for the recruitment of XPC to UV-damaged sites in DNA [43]. Another component of the NER pathway indispensable for the removal of damaged DNA fragments is TFIIH (transcription factor II H). It is a ssDNA (single-stranded DNA) binding multi-subunit complex made up of kinase and core subcomplexes. The kinase subcomplex—CAK (CDK (cyclin-dependent kinase)-activating kinase) of TFIIH contains CDK7, MAT1 (CDK-activating kinase assembly factor MAT1) and cyclin H, whereas the core subcomplex consists of XPB, XPD, p62, p52, p44, p34, and p8 (general transcription factor IIH subunit 5) (Figure 2d). Interaction of XPC with XPB and/or p62 is necessary for the recruitment of TFIIH to the damaged site [44,45]. MAT1, a component of the CAK subcomplex, was shown to interact and inhibit the helicase activity of XPD. This effect was relieved in the presence of the p44 core subunit [46]. However, results from other studies have indicated that the TFIIH core is enough to support in vivo GGR without the contribution of CAK activity [47]. TFIIH functioning is regulated by the XPA protein. This protein has significantly higher affinity to specified DNA lesions/incorrect structures than to undamaged DNA [48,49]. XPA was demonstrated to be recruited to TFIIH in vitro leading to CAK dissociation (Figure 2e) [50]. XPB and XPD (components of the TFIIH core complex) are DNA helicases that promote DNA opening [51]. This NER step is dependent on ATPase, but not on helicase activity of XPB and on both ATPase and helicase activities of XPD [52]. Whereas XPA stimulates helicase activity of the TFIIH core complex in undamaged DNA, it inhibits the TFIIH core complex translocation along the DNA strand when a DNA lesion is detected [53]. The above findings were summarized in a recent model presented by Kusakabe and co-workers [54] where following the recruitment of TFIIH to XPC-HR23-CEN2, the XPD helicase in conjunction with XPA scan the DNA strand in the 5′-3′ direction. The presence of a lesion in DNA blocks the translocation of the XPA-TFIIH complex [54]. Cryo-EM studies support this model by showing that XPA contributes to the recognition of the 5′ end of the DNA repair bubble [55]. Subsequently, a DNA lesion is verified by TFIIH and XPA. The release of the XPC-HR23-CEN2 complex involved in the initial recognition of DNA damage occurs simultaneously with the recruitment of XPG and XPF-ERCC1 (excision repair cross-complementation group 1) to TFIIH (Figure 2j) [56]. GGR studies on a yeast model indicated that RAD2 (a yeast homolog of human XPG) competes with RAD4 (a yeast homolog of human XPC) for binding sites on TFIIH [57]. Recently, XPA and XPG have been proved to stimulate DNA unwinding activity of XPD in vitro by around 20-fold [55]. The nuclease activity of XPG and XPF-ERCC1 acting at 3′ and 5′ sides of DNA damage, respectively are necessary for the incision of a DNA fragment containing a lesion [58,59,60]. It results in the removal of a damaged ~30 nucleotides DNA fragment [61,62]. The filling of an emerging gap is dependent on coordinated action of the many enzymes involved in the DNA metabolism including proteins such as pol *δ*, pol *ε*, PCNA (proliferating cell nuclear antigen), DNA LIG1 (DNA ligase 1), RFC (replication factor C), RPA [63], XRCC1 (X-ray repair cross-complementing protein 1) and DNA LIG3 [64].

The scenario of DNA damage recognition during TCR differs from the one described for GGR. It is believed that in TCR, RNAPII (RNA polymerase II) plays the role of a DNA lesion sensor in a transcribed DNA strand [65]. RNAPII stalled at the lesion (RNAPIIo) serves as a signal for the recruitment of other proteins necessary for DNA repair (Figure 2f). It is thought that TCR is initiated by RNAPIIo-bound CSB (Cockayne syndrome, Group B), which recruits CSA (Cockayne syndrome, Group A). CSA facilitates the association of stalled RNAPII with UVSSA (UV-stimulated scaffold protein A) (Figure 2g). UVSSA recruits TFIIH through interaction with a component of its core subcomplex, p62 [66,67,68,69]. Recent studies on a murine model have shown that the CSA-CSB complex facilitates cullin-ring type E3 ligase-mediated ubiquitination of RPB1 (RNA polymerase II subunit B1), a subunit of RNAPII (Figure 2g). Together with sequential ubiquitination of UVSSA it was found to stimulate the association of the core TFIIH complex with RNAPIIo (Figure 2h). This seems to be essential for transcription recovery and repair of UV-induced lesions [69]. Although the proteins necessary for TFIIH recruitment differ for GGR and TCR, the TFIIH recruitment mechanism and the subsequent steps are common for both subpathways (Figure 2d,e,h–j) [67,70].

Homologs of most human and yeast genes which encode proteins related to GGR and TCR have been found in plants [71,72]. However, a homolog of the gene coding for XPA, a key protein in human and yeast NER, has never been found in plants. Whether another plant protein plays a role similar to XPA, and whether lesion recognition and regulation of TFIIH activity during NER in plants are similar to these processes in humans and yeasts remain open questions.

In plant cells, similarly to yeast and human cells, NER plays an important role in the repair of UV-induced DNA damage. The involvement of some Arabidopsis homologs of yeast and human GGR proteins in plant response to UV has been demonstrated. The Arabidopsis *atuv-ddb2, ddb1a* and *ddb2* mutants were hypersensitive to UV [73,74]. Repair of CPDs and 6-4 PPs in plants with mutation in *AtDDB1A* gene was less effective comparing to wild type (WT) Arabidopsis. Moreover, *AtDDB2* gene mutants were shown to be defective in SSD (synthesis-dependent DNA) repair while plants overexpressing *AtDDB1A* removed CPDs and 6-4 PPs more efficiently [74,75]. AtDDB2 was proposed to cooperate with AtDET1 (de-etiolated1) which is necessary for efficient removal of UV-induced photoproducts through NER pathways [76].

Plants deficient in AtCEN2 displayed reduced repair efficiency of UV-damaged DNA and higher frequency of homologous recombination [77]. In contrast to the human and yeast genome, the Arabidopsis genome encodes two isoforms of the XPB protein called AtXPB1 and AtXPB2. Both AtXPB1 and AtXPB2 partially complemented UV resistance of the yeast *rad25* mutant with an impaired gene homologous to the human *XPB* [78,79]. Another protein of the NER system investigated in Arabidopsis is a homolog of the human XPD. Whereas the Arabidopsis knock-out mutant of *AtXPD/AtUVH6* (ultraviolet hypersensitive 6) gene is lethal, the *uvh6-1* mutant plants carrying a point mutation in this gene were found to have only mild growth defects and reduced excision of 6-4 PPs resulting in increased sensitivity to UV [80]. AtUVH3/UVR1 (ultraviolet hypersensitive 3/uv repair defective 1) is a homolog of the human XPG/*Saccharomyces cerevisiae* RAD2 nuclease involved in NER [81]. Dark repair of 6-4 PPs was not observed in the *uvh3-1* mutant even 24h after UV-C exposure when about 55% of these lesions had been repaired in WT plants. This result indicates a crucial role of AtUVH3 in the dark repair of 6-4 PPs [80]. The Arabidopsis *uvh1* mutant deficient in AtRAD1/UVH1, a homolog of the human XPF and yeast RAD1, demonstrated hypersensitivity to UV [82]. *AtRAD1* antisense plants were impaired in dark repair of CPDs [83]. Moreover, when expressed in the *Saccharomyces pombe rad16* mutant, AtRAD1 partially reduced yeast sensitivity to UV. An analysis of the *uvr7–1* (lacking the human ERCC1 homolog) and the *cul4* Arabidopsis mutants indicated that impaired genes play significant function(s) in plant response to UV light [84]. Surprisingly, a human ERCC1 homolog from *Lilium longiflorum* reduced the hypersensitivity of *ERCC1*-deficient Chinese hamster ovary cells to mitomycin C but not to UV [85].

Enhanced removal of CPDs from a transcribed strand as compared to a non-transcribed strand indicates the presence of TCR in plants [86]. This finding complies with previous results demonstrating that Arabidopsis *CSA*-like genes, *AtCSA-1 and AtCSA-2* (also referred to as *CSAat1A* and *CSAat1B*)*,* regulate plant response to UV [87]. Diminished repair of thymidine dimers was detected under dark conditions in an Arabidopsis mutant with an impaired AtCSA-1 function indicating its key role in dark repair [88]. In vivo studies using the Arabidopsis T87 cell line demonstrated that dark repair of CPDs and 6-4 PPs in plants occurs by a dual-incision mechanism identical to the one found in other eukaryotes [89]. Furthermore, TCR of UV-induced DNA lesions in Arabidopsis was proposed to be controlled by joint actions of the circadian clock and transcription performed by RNA polymerase II [90]. The authors reported that dark repair of CPDs in the Arabidopsis genome is determined by three main factors: transcription, the circadian clock and chromatin state. The preferred repair of the transcribed strands of active genes allows a rhythmic, coordinated repair of genes belonging to the same biochemical trait. Recently, increased sensitivity of the Arabidopsis *atcsa-1, uvssa-2, ubp12, rdo2* and *chr8-2* mutants, with impaired functions of genes coding homologs of human proteins involved in NER, to UV was demonstrated [91,92]. Khateeb and co-workers [92] proposed that AtUBP12 (ubiquitin specific protease 12) is involved in the deubiquitination of UVSSA, however no experimental data confirming this interpretation has been shown. This suggestion is intriguing since UBP12 is known to be involved in the deubiquitination of human and yeast DDB2 protein [93]. AtRDO2 (reduced dormancy 2) is a homolog of the human TFIIS (transcription elongation factor TFIIS) involved in the restart of RNAPII arrested at a DNA lesion [94]. The growth of hypocotyl and/or root of *atcsa-1, uvssa-2, ubp12, rdo2* but not of *chr8-2* plants was significantly affected only when the plants were kept in darkness after UV treatment. The growth of the *chr8-2* mutant was substantially delayed after UV irradiation regardless of the light conditions. This suggests that the Arabidopsis homolog of the human CSB encoded by *AtCHR8* may play a role in both dark and light DNA repair. Studies of the *atpolλ* mutant plants revealed inhibited germination and seedling growth after UV-B treatment. These plants displayed slower repair of UV-B induced CPDs in the dark. The involvement of the AtPOL*λ* protein in the removal of CPD by the NER pathway was shown also in vitro [95].

Up to date, the presence of NER, an indisputably essential element of the nuclear DNA repair machinery in eukaryotes, has been reported neither in plant chloroplasts nor in the mitochondria [96].

## 4. Base Excision Repair

**Figure 3:**
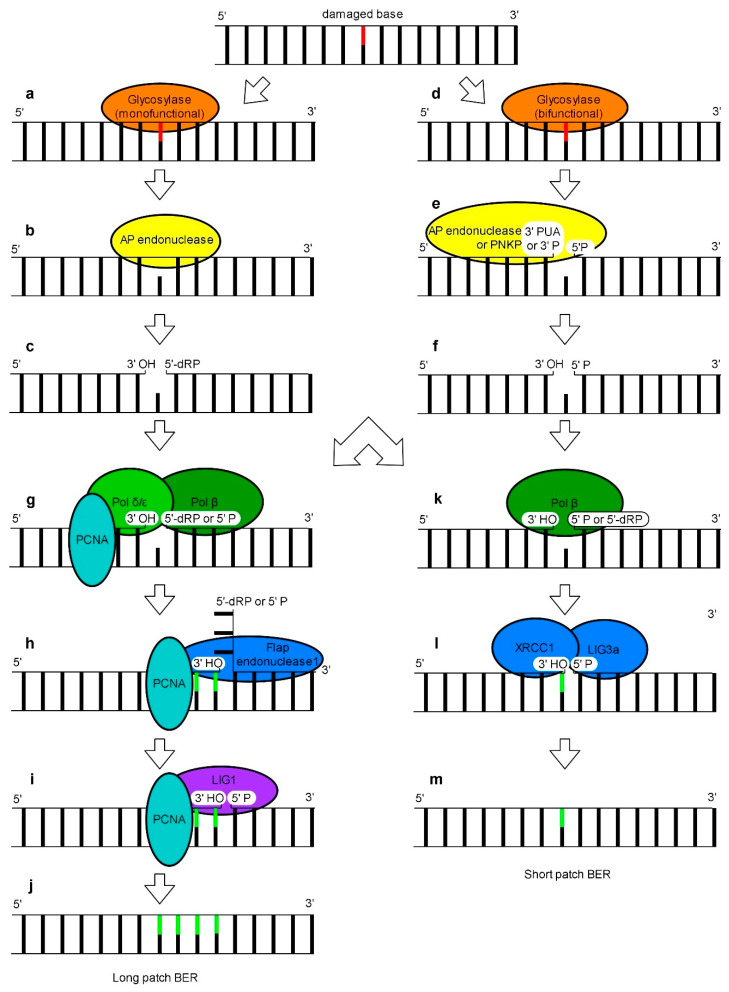
A schematic representation of the long patch BER and short patch BER mechanism based on a mammalian model. In BER, the recognition and removal of a damaged base by a monofunctional glycosylase (**a**) results in the formation of an apurynic/apyrimidinic (AP) site (**b**)**.** Next, an AP endonuclease cleaves a sugar backbone resulting in free 3′OH and 5′-dRP termini (**c**). In an alternative scenario, a bifunctional glycosylase (**d**) forms a Schiff base and cleaves a sugar backbone leading to the formation of 3’-PUA or 3’-P and 5’-P termini (**e**) followed by the conversion of 3’-PUA or 3’P to 3′OH by an AP endonuclease or phosphatase (PNKP (polynucleotide kinase 3’-phosphatase) in mammals), respectively (**f**). Products of reactions initiated by mono- and bifunctional glycosylases can undergo repair by the LP-BER (**g**–**j**) or SP-BER (**k**–**m**) subpathway. In LP-BER, where the conversion of 5′-dRP to 5′-P terminus is not necessary, pol *β* inserts the first nucleotide while the following ones are incorporated by pol *δ* and pol *ε* in cooperation with PCNA (**g**). Displaced nucleotides form a flap structure which is cleaved by FEN1 (**h**). Finally LIG1 ligates the adjacent 3’ and 5’ ends of a resynthesized and nicked DNA fragment (**i**). The repaired DNA has between 3-10 resynthesized nucleotides (**j**). In SP-BER, pol *β* converts 5’-dRP generated by a monofunctional glycosylase to 5’-P terminus. Moreover, it fills one nucleotide gap generated by an AP endonuclease or a bifunctional glycosylase in cooperation with an AP endonuclease (**k**). It is proposed that in plants pol *λ* may play the role of pol *β*. The XRCC1 and LIG3a complex ligates 3’ OH of an inserted nucleotide with 5’-P of a DNA backbone (**l**). As the result only one, damaged, nucleotide in a DNA strand is replaced (**m**).

Among various types of DNA damage caused by UV radiation, modified that is oxidized or deaminated bases may be observed [10,97]. One of the repair systems that can remove these lesions is BER (Figure 3). The BER mechanism operating in eukaryotes on nuclear DNA is relatively well understood. In the first step, a DNA glycosylase recognizes a damaged base [98]. DNA glycosylases differ in substrate specificity and in catalytic power allowing living organisms to cope with different types of base lesions [99,100]. DNA glycosylases might be either monofunctional or bifunctional enzymes carrying additionally AP (apurynic/apyrimidinic) lyase activity [101]. Upon detection of a damaged base, monofunctional DNA glycosylases cleave an N-glycosidic bond which results in the release of the damaged base and the formation of an AP site i.e., an intact sugar-phosphate backbone (Figure 3a) [102]. Next, an AP endonuclease cleaves a sugar backbone at the 5′ end of an abasic site giving free 3′-OH and 5′-dRP (5′ deoxyribose 5-phosphate) termini (Figure 3c). When bifunctional DNA glycosylases process the damaged site, in the first step a Schiff base intermediate is formed. Subsequently, internal AP lyase activity of these enzymes cleaves a DNA backbone at the 3′ site of the DNA lesion by *β*-elimination, leading to the formation of 3′-PUA (3′-*α*, *β* unsaturated aldehyde), and 5′-P (5′-phosphate) termini (Figure 3b). Next, 3′phosphodiesterase activity of AP endonucleases converts a 3′-PUA end into a free 3′-OH’ (3′ hydroxyl) terminus (Figure 3d). Some bifunctional glycosylases convert the 3′-PUA end into a 3′-P (3′-phosphate) by a *δ*-elimination reaction. Subsequently, DNA 3′-phosphatases eliminate a phosphate group from a 3′-P terminus which results in the formation of a free 3′OH end. In mammalian cells this reaction is carried out by PNKP (polynucleotide kinase 3′-phosphatase) (Figure 3d) [103,104]. In consequence, monofunctional and bifunctional DNA glycosylases excise the lesion producing single-strand breaks with a 3′-OH’ terminus and either 5′-dRP or 5′-P terminus, respectively (Figure 3e,i). To complete the DNA repair process, the remaining gap must be filled via one of the two alternative BER subpathways called SP (short patch) and LP (long patch) [105]. BER studies on a mammalian cell model revealed that a DNA glycosylase involved in DNA damage recognition and removal determines which BER subpathway is used [106]. SP BER can operate on substrates with a free 5′-P group. Therefore, mammalian cells use pol *β* (DNA polymerase *β*) to convert non-functional intermediate BER products with the remaining 5′-dRP, generated by monofunctional glycosylases, to functional ones with a 5′ phosphate group at undamaged nucleotides. This polymerase carries a 5′-dRP lyase activity [107,108]. Pol *β* also fills one nucleotide gap generated during the first stages of BER (Figure 3i). Next, the XRCC1-LIG3a (DNA ligase 3a) complex is recruited to ligate a 3′-OH group of inserted nucleotides with a 5′-OH group of a DNA backbone carrying phosphate (Figure 3j) [109,110]. Interestingly, incorporation of the first nucleotide in LP BER is also dependent on pol *β* (Figure 3e) [111]. In this subpathway, the removal of 5′-dRP from an intermediate BER product generated by monofunctional glycosylase is possible but not indispensable. The elongation of a DNA strand is continued by replicative pol *δ* and pol *ε* which displace the downstream strand carrying the 5′-dRP moiety leading to the formation of a flap structure (Figure 3e). Subsequently, the flap is cleaved by FEN1 (flap endonuclease 1) (Figure 3f) and finally the new DNA fragment and the “old” DNA strand with a free 5′-P terminus, generated by cutting off the flap structure, are ligated by LIG1 (Figure 3g) [104]. Successful completion of LP BER is dependent on the presence of PCNA, a pol *δ* processivity factor, which is loaded onto DNA by the RFC complex. The role of PCNA in LP-BER is to coordinate the synthesis of a new DNA strand, flap digestion and ligation of adjacent 3′ and 5′ ends of DNA (Figure 3e–g) [17]. Recently, a new branch of LP BER called 5′ gap-mediated LP-BER has been found. After the abasic site excision by AP endonuclease, the PARP1 (poly(ADP-ribose) polymerase)-RECQ1(ATP-dependent DNA helicase Q-like 1)-RPA complex produces an 8-nucleotide 3′ flap which is excised by ERCC1-XPF. This eventually results in a 9-nucleotide gap at the 5′ termini of the lesion [112]. The subsequent filling of this gap has been proposed to be similar to the classical LP BER. 

Plants have been shown to possess homologs of proteins assigned to the BER pathway in animal and yeast cells. The currently available data indicate that in addition to nucleus, BER operates also in plant mitochondria and chloroplasts [96,113]. Activity of UNG (uracil DNA glycosylase), engaged in BER of uracil, a product of cytosine deamination, was observed in maize nuclear fraction and mitochondria of Arabidopsis, maize and potato [114,115,116]. Moreover, in vivo studies of AtUNG revealed targeting of this protein to mitochondria [115]. This is of high importance since studies on *E. coli* have shown that the UV-induced formation of pyrimidine dimers in DNA increases the rate of cytosine deamination by six orders of magnitude which causes the appearance of uracil in a DNA strand. This leads to accelerated formation of single C→T and tandem double CC→TT mutations in a genome [10]. In Arabidopsis several genes coding for putative nuclear DNA glycosylases have been found. In vitro experiments indicated that AtMMH (MutM homolog) [117], AtFPG1 (formamidopyrimidine-DNA glycosylase; [118]) and AtOGG1 (N-glycosylase/DNA lyase OGG1; [6,119]) possess functions which can repair 8-oxo-dG, typical oxidative damage induced by UV. In line with this, inactivation of *AtFPG* and *AtOGG1* genes results in higher incidence of oxidative DNA lesions in nuclear and mitochondrial genomes [120]. The product of a reaction catalyzed by DNA glycosylases AtFPG and AtOGG1 may serve as a substrate for polynucleotide 3’-phosphatase AtZDP (polynucleotide 3’-phosphatase ZDP) and AtARP (DNA-(apurinic or apyrimidinic site) endonuclease) which is the major source of AP endonuclease activity in Arabidopsis cell extracts [120,121]. Extremely low activity of OGG1 detected in potato mitochondria was also presented [122]. However, it should be treated with caution. It may be a result of contamination of mitochondrial fraction with nuclear proteins. Studies of Arabidopsis *fpg* and *ogg1* double, but not single, mutants demonstrated an increase in oxidative damage of both nuclear and mitochondrial DNA. This suggests that activities of these glycosylases can compensate each other [120]. Glycosylase-lyase/endonuclease activity was absent in chloroplast protein extracts prepared from Arabidopsis *atarp* mutants which confirms organelle targeting of BER repair proteins apart from the nucleus [113]. Chloroplast DNA glycosylase AtNTH1 (endonuclease III homolog 1) has been shown to incise a UV-irradiated supercoiled DNA but not undamaged DNA [123]. Activity of AP endonuclease was confirmed in mitochondria of Arabidopsis, potato and *Araucaria angustifolia* [115,122,124]. 

Apart from DNA glycosylases and AP endonucleases, also genes coding other components essential for BER and other DNA repair pathways were found in plants including DNA pols, AtLIG1, AtPCNA1, AtPCNA2 and AtFEN1 [17,104]. Among these proteins AtFEN1 was shown to play a role in plant response to UV. The single point mutation in the *AtFEN1* gene which affects its splicing efficiency causes hypersensitivity of *sav6* mutant seedlings to UV-C. In addition to less abundant full-length AtFEN1 compared to WT plants, a truncated protein is produced in the mutant [125]. Although plants lack homologs of human pol *β* and LIG3a, both SP BER, alternatively called SN (single nucleotide) BER, and LP BER pathways are active in plants [121]. It has been speculated that AtPOL*λ*, which has at least in vitro dRP-lyase activity*,* may potentially play a pol *β*-like role in plant BER pathway [126]. To complete BER process in mitochondria and chloroplasts activity of DNA polymerase and ligase is indispensable. The presence of two DNA polymerases, AtPOL IA and AtPOL IB, targeted to both mitochondria and chloroplasts of Arabidopsis and tobacco was reported [127,128,129]. These enzymes showed 5′-dRP lyase activities necessary for removal of the 5′-dRP formed during SP BER. Whereas, AtPOL IB performed efficient strand-displacement on DNA containing a one or two-nucleotide gap, AtPOL IA was less efficient in strand-displacement. It was therefore suggested that both these polymerases can be involved in SP-BER, while only AtPOL IB was proposed to be involved in LP BER [130]. One of AtLIG1 isoforms was shown to be targeted exclusively to Arabidopsis mitochondria [131]. The mitochondrial or chloroplast 5′-flap endonuclease(s) and chloroplast DNA ligase involved in BER have not been identified in plants so far. 

## 5. Mismatch Repair

**Figure 4:**
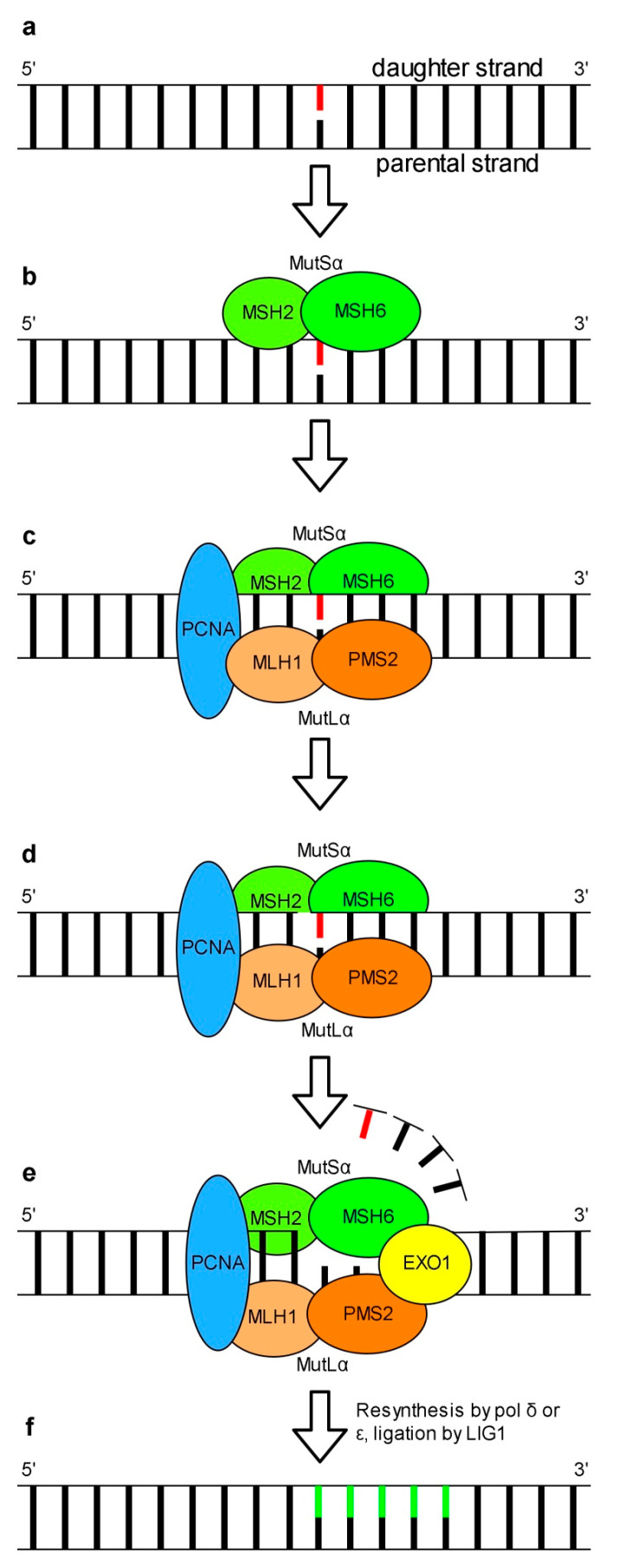
A scheme presenting the repair of mismatched nucleotides by MMR based on a mammalian model. A mismatched pair of nucleotides at complementary DNA strands does not create correct hydrogen bonds (**a**). The MutS*α* complex, composed of MSH2 and MSH6 recognizes unpaired bases (**b**). MutS*α* interacts with MutL*α* made up of MLH1 and PMS2. The MutS*α*-MutL*α*-DNA complex interacts with PCNA (**c**). PCNA activates MutL*α* attenuated endonuclease activity, which cleaves the DNA strand (**d**). 5′-3′ exonuclease of activity of EXO1, activated by MutS*α* or MutL*α* bound to the lesion, removes the DNA fragment containing incorrect nucleotide (**e**). Resynthesis of a missing DNA strand performed by pol *δ* or pol *ε* and assisted by PCNA is followed by its ligation performed by LIG1 (**f**).

Mismatched nucleotides in opposed DNA strands are among various abnormalities in genetic material whose propagation can result in serious consequences for proper cell functioning. This type of errors results from incorporation of an inappropriate nucleotide or its tautomeric form, nucleotide insertion or deletion by replicative pols. The role of MMR in the repair of UV-induced DNA damage is unclear. Experimental data indicates that MMR does not repair UV-induced DNA lesions but rather contributes to the suppression of mutagenesis of nuclear DNA (reviewed: [132]). Data published by Tsaalbi-Shtylik and co-workers [133] reveals that MMR in eukaryotes is engaged in the excision of inappropriate nucleotides incorporated opposite to DNA photolesions during TLS [133]. The detection and repair of mismatches in human nuclear DNA is dependent on either the MutS*α* or MutS*β* complex. MutS*α* which recognizes 1-2 unpaired base(s) is made up of MSH2 (MutS homolog 2) and MSH6 proteins which form a clamp-like structure that detects and binds mismatched nucleotides (Figure 4b). MutS*β* consisting of MSH2 and MSH3, detects insertion-deletion loops of 1-15 nucleotides as well as DNA with 3′ single-stranded overhangs (reviewed: [134]). MutS*α* has two ATP binding sites [135,136]. The current model of mammalian MutS*α*-dependent MMR assumes that recognition of a DNA mismatch by MutS*α* is followed by ATP binding. This leads to conformational changes of MutS*α* and promotes its interaction with the MutL*α* complex made up of MLH1 (MutL protein homolog 1) and PMS2 (PMS1 protein homolog 2) (Figure 4c) [137]. The MutS*α*-MutL*α*-DNA complex interacts with PCNA (Figure 4c). The role of PCNA in MMR is the activation of attenuated MutL*α* endonuclease activity necessary for the incision of a daughter DNA strand in the region carrying an error (Figure 4d) [138,139]. After DNA strand nicking at the 5′ end of the mismatch by MutL*α* a DNA fragment containing an inappropriate nucleotide is removed by 5′-3′ exonuclease activity of EXO1 (exonuclease 1). EXO1 is activated by MutS*α* or MutL*α* bound to the lesion (Figure 4e) [140,141]. Resynthesis of the missing DNA strand is performed by either pol *δ* or pol *ε* (Figure 4f). Finally, a newly synthetized DNA fragment is ligated with a DNA backbone by LIG1 (Figure 4f) [142]. Alternatively, instead of the removal of a DNA fragment carrying the lesion by EXO1, a strand displacement synthesis mediated by pol *δ* or pol *ε* to a position behind the mispair occurs [143]. Details of models of EXO1-dependent and -independent MMR can be found in a review of Goellner and co-workers [144].

Homologs of most mammalian genes involved in MMR have been identified in the plant genomes. Interestingly, the *MSH7* gene encoding a protein which forms part of this DNA repair pathway is unique for plants [145]. Arabidopsis AtMSH2 forms heterodimers with either AtMSH6, AtMSH3 or AtMSH7. These heterodimers are called MutS*α*, MutS*β* and MutS*γ*, respectively and they differ in the affinity/preference to particular DNA substrates. Specificities of plant MutS*α* and MutS*β* against selected mismatched substrates are similar to their homologs found in other eukaryotes. MutS*γ* which is unique for plants, can recognize additional lesions. The presence of three different MutS complexes enables plants to recognize a wide spectrum of mismatches [145,146]. Upregulation of the *MSH2* and *MSH6* gene expression upon UV-B irradiation in Arabidopsis and maize indicates the involvement of MMR in plant response to UV [147]. The levels of CDPs in T-DNA mutants of the *AtMSH2, AtMSH6* and *AtMSH7* genes were higher than in WT plants when irradiated with UV-B followed by irradiation with UV-A promoting repair of CPDs [147,148]. This implies the role of AtMSH2*,* AtMSH6 and AtMSH7 in the repair of CPDs. As mentioned above, mismatches in DNA result among others from errors in DNA replication. Whereas the genes involved in dark repair are predominantly expressed in proliferating plant cells, the expression of the *AtMSH* genes was reported also in mature leaves [18]. Therefore, the putative role of MMR in the control of genome instability induced by environmental factors in non-dividing plant tissues is one of open questions.

The only known plant MMR protein with demonstrated mitochondrial and plastid targeting is AtMSH1. Mutants with AtMSH1 deficiency showed enhanced recombination of mitochondrial and plastid genomes. Suppression of *MSH1* expression by RNAi resulted in a variegation phenotype in Arabidopsis, tomato, tobacco, pearl millet and sorghum. Deficiency of *AtMSH1* caused incomplete development or premature degeneration of plastids [149]. It has been shown recently that the disruption of the *At*MSH1** gene caused a 10-fold and 1000-fold increase in the mutation frequency in mitochondrial and plastid genomes, respectively, indicating an essential role of the encoded protein in controlling the rate of organellar DNA mutagenesis [150]. Finally, mismatched nucleotides in chloroplast can be repaired by gene conversion [151].

## 6. Repair of DNA Breaks

SSBs are discontinuities in one strand of the DNA double helix. Among the factors that lead to the formation of SSBs are UV-induced ROS (reactive oxygen species). Direct generation of SSBs may result from disintegration of oxidized sugar in a DNA backbone. In addition, SSBs may be formed indirectly during the BER of damaged bases, e.g., oxidized or abasic sites (reviewed: [152]). Unrepaired SSBs in proliferating cells cause stalling of DNA replication forks which may lead to their collapse and eventually to the formation of DSBs [153]. DSBs are highly deleterious DNA lesions [154]. Eukaryotes use different mechanisms to prevent the accumulation of DSBs (reviewed: [155,156,157]). ATM (ataxia-telangiectasia mutated), ATR (ATM- and Rad3-related) and DNA-PKcs (DNA dependent protein kinase catalytic subunit) kinases play the role of primary sensors and signal transducers of DNA breaks. ATM is activated and recruited to DSBs by the MRN complex composed of MRE11, RAD50 and NBS1 (Nijmegen breakage syndrome 1) proteins. ATR is recruited to RPA-coated ssDNA by ATRIP. DNA-PKcs is recruited and activated by Ku70/80-bound DSB ends. ATM, ATR, and DNA-PKcs kinases phosphorylate downstream signal transducers, regulators and effectors of DNA damage repair pathways including histone H2AX [158]. Thus, phosphorylated H2AX (*γ*-H2AX) is a marker of DNA damage [159,160]. It plays a key role in the recruitment and the accumulation of DNA repair proteins at the sites of a damage such as DSB (reviewed: [161]). Besides ATM, ATR and DNA-PKcs other proteins including MDC1 (mediator of DNA-damage checkpoint protein 1) and 53BP1 (p53-binding protein 1), play also important role in regulation of DNA damage response to DSBs. MDC1 cooperating with *γ*-H2AX and other factors controls amplification of *γ*-H2AX signal while 53BP1 channels DSB repair toward NHEJ [158,162,163].

Most SSBs are repaired by a rapid global SSB repair process. The recognition and processing of damaged base by DNA glycosylase followed by the action of AP endonuclease results in indirect formation of intermediate SSBs which are processed by BER proteins acting downstream (see section devoted to BER). The recognition of directly formed SSBs, independent of the processes described above, requires the presence of PARP1 (reviewed: [152]). Upon SSBs binding PARP1 is activated and undergoes poly ADP-ribosylation which is indispensable for the recruitment of other BER proteins [164]. Homologs of human ATM, ATR and PARP1 including Arabidopsis AtPARP1 and AtPARP2 have been identified in plants. They were proposed to be engaged in DNA repair although biochemical studies of these proteins have not been reported yet [165,166,167]. 

In the case of eukaryotes, DSBs are repaired by two major pathways, NHEJ and HR. In plant somatic cells they are preferentially repaired by NHEJ [155].

### Repair of DSBs by Non-Homologous End Joining and Homologous Recombination

**Figure 5:**
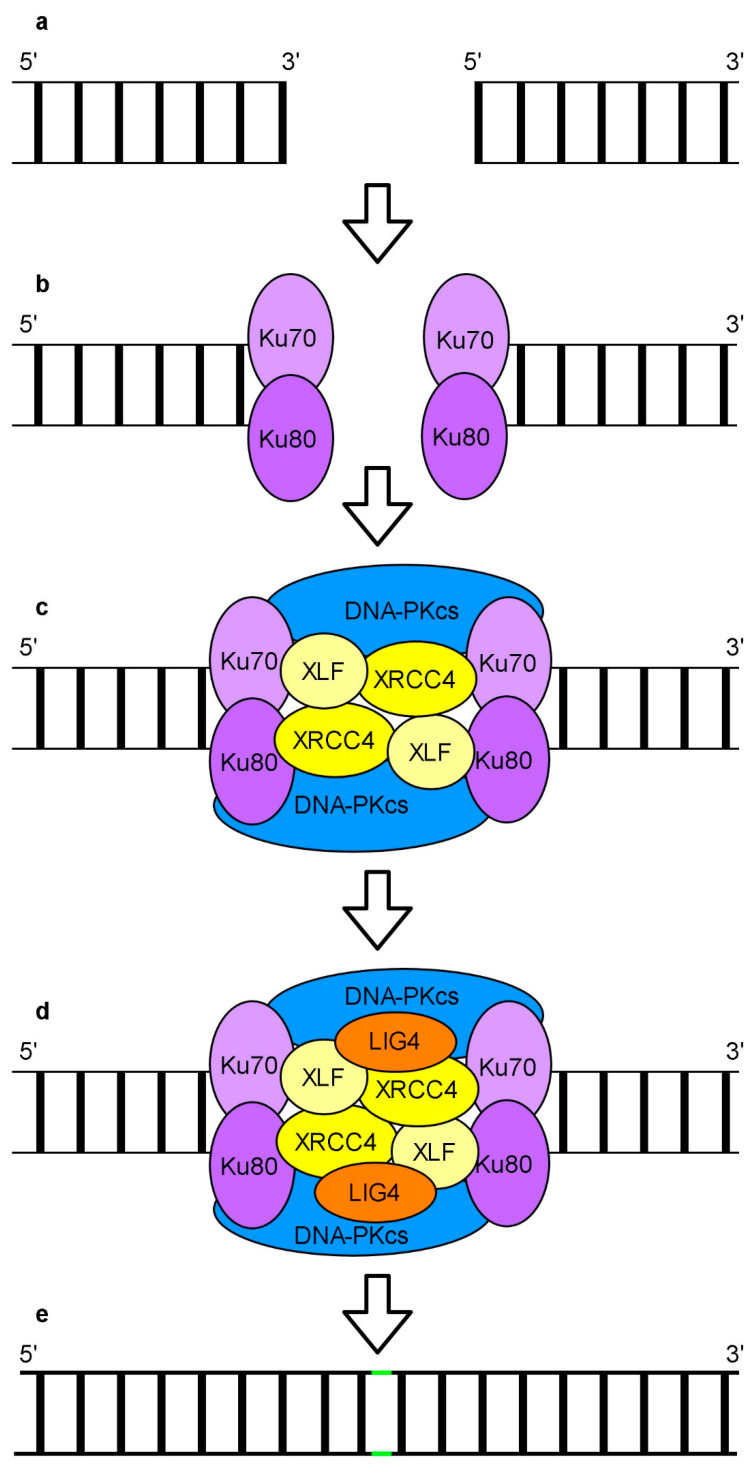
A schematic depiction of canonical NHEJ based on a mammalian model. An example of double-strand breaks (DSBs) with two blunt ends (**a**), which interact with Ku70/80 complexes (**b**). Ku70/80 recruits DNA-PKcs (DNA dependent protein kinase catalytic subunit) as well as XRCC4 (x-ray cross complementing protein 4) and XLF (XRCC4-like factor), which create a filament between two loose DNA ends (**c**). LIG4 is recruited to ligate the DNA ends (**d**). Ligated DSB (**e**).

NHEJ is a leading error prone repair system of DSBs in the nuclei of higher eukaryotes (Figure 5) (reviewed: [157,168,169,170,171,172,173]). 

A NHEJ major subpathway, called cNHEJ (canonical), is dependent on the Ku70/80 (ATP-dependent DNA helicase Ku70/80) heterodimer (Figure 5b). Ku70/80 recognizes and binds to DNA termini recruiting other core factors of cNHEJ such as DNA-PKcs, XRCC4 (x-ray cross complementing protein 4), XLF (XRCC4-like factor) and LIG4 (DNA ligase 4). DNA-PKcs activity is stimulated by DNA bound Ku70/80. DNA-PKcs kinase regulates the function of other proteins involved in cNHEJ. XRCC4 and XLF proteins form a filament which can bridge both ends of broken DNA (Figure 5c). It is assumed that cooperation of this filament with DNA-PKcs and Ku70/80 results in the formation of a complex responsible for the protection of DNA termini. The nature and complexity of DSBs determine the proteins involved in subsequent steps of DNA repair. A scaffold formed by the XRCC4 protein may recruit specific DNA end processing enzymes including PNKP, APTX (aprataxin), APLF (aprataxin and PNKP-like factor), TDT (terminal deoxynucleotidyl transferase) and Artemis nuclease. These enzymes are responsible for the processing of DSBs to create ligatable DNA ends. A mammalian WNR (Werner) protein was shown not only to cooperate with the XRCC4-LIG4 complex in end-processing [174] but also to promote cNHEJ and inhibit other NHEJ subpathways [175]. To fill gaps in complex DSBs, pol *λ* or pol *μ* are used. The final step of cNHEJ is DNA end ligation performed by LIG4 whose activity is stimulated and regulated by XRCC4 (Figure 5d). Apart from the Ku70/80 dependent cNHEJ pathway, other ncNHEJ (non-canonical NHEJ) pathways such as bNHEJ (backup NHEJ), dependent among the others on XRCC1, and MMEJ (microhomology mediated end joining), requiring a microhomologous sequence flanking junction of the DSB site, have been described. In both cases, the PARP1 rather than Ku70/80 complex recognizes and binds DSBs. 

**Figure 6:**
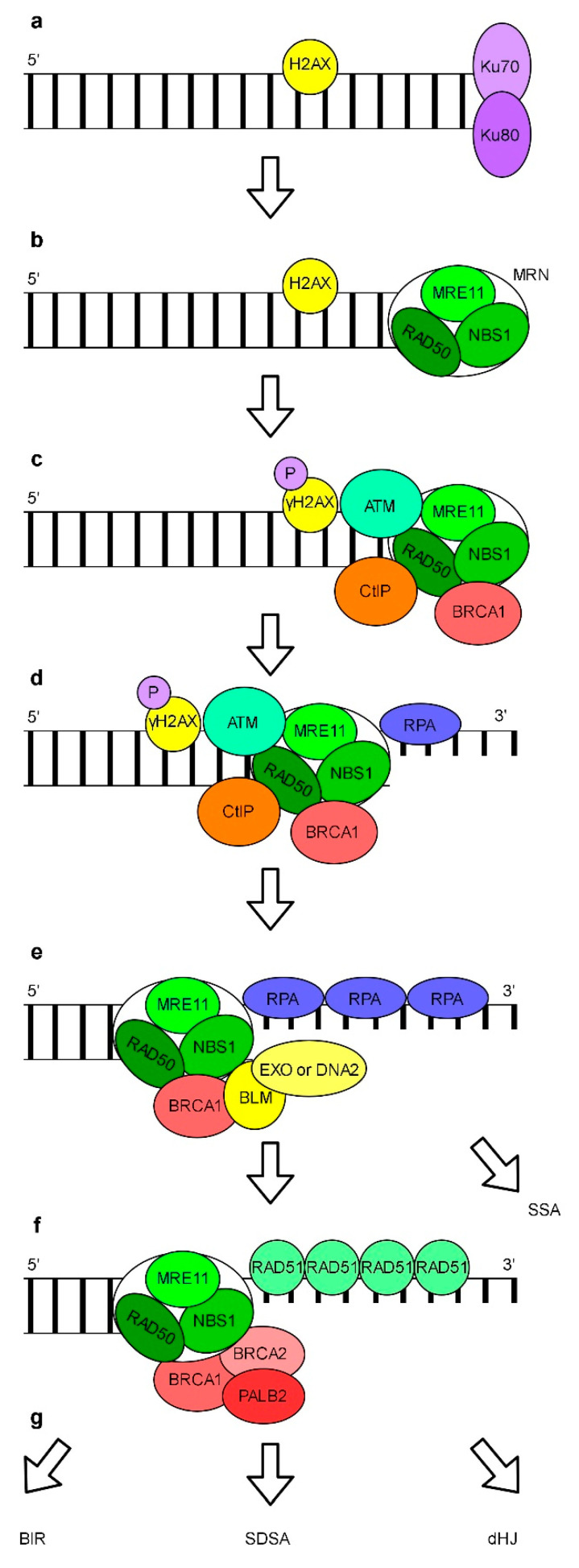
A simplified mechanism of homologous recombination based on a mammalian model. The DNA termini of DSBs are coated by the Ku70/80 complex (**a**). The MRN complex, composed of MRE11, RAD50 and NBS1 replaces Ku70/80 (**b**). MRN recruits ataxia-telangiectasia mutated (ATM), which phosphorylates histone H2AX positioned close to the site of a break. BRCA1 and CtIP are recruited (**c**). MRN with BRCA1 and CtIP promote a short resection of the free 5′ DNA end creating a 3′ overhang, which is coated by RPA (**d**). BLM and EXO1 or DNA2 are recruited and generate a longer 3′ overhang which is coated with RPA (**e**). The BRCA1-PALB2-BRCA2 complex promotes a replacement of RPA by RAD51 monomers and the formation of a RAD51 filament (**f**). The repair is completed by the BIR (break-induced replication), SDSA (synthesis-dependent strand annealing) or dHJ (double Holliday junctions) pathways (**g**) and alternatively by the SSA (single-strand annealing) pathway after the resection of DNA by BLM and EXO1 or DNA2 (**e**).

In addition to NHEJ, DSBs may be repaired by an error-free HR pathway (Figure 6). The HR usually does not result in a sequence loss which ensures the stability of genomic information. The HR pathway interferes with cNHEJ. The current model assumes that successful initiation of HR is dependent on the removal of Ku70/80 proteins from DNA ends (Figure 6a). Human RAD17, a replication checkpoint protein, is required for early recruitment of the MRN (MRE11-RAD50-NBS1) complex to the DSB site (Figure 6b) [176]. A multifunctional MRN complex binds to DSB and recruits ATM which phosphorylates histone H2AX close to the site of a DNA break (Figure 6c). This initiates a cascade of chromatin modifications. MRN, BRCA1 (breast cancer type 1 susceptibility protein) and CtIP (C-terminal binding protein 1 (CtBP1) interacting protein) promotes a short resection of free 5′ DNA ends which results in the formation of 3′ overhangs (Figure 6d). In the following step, the BLM (Bloom’s syndrome) helicase and either the DNA2 (the DNA replication helicase/nuclease 2) nuclease or EXO1 are recruited and larger 3′ protruding ends are generated and rapidly coated with RPA (Figure 6e) [177]. Subsequently, RPA is displaced by RAD51. This requires the presence of the BRCA1-PALB2-(a partner and localizer of BRCA2)-BRCA2 complex which promotes the disassembly of RAD51 heptamers, loading of monomeric RAD51 onto ssDNA and the formation of the RAD51 filament (Figure 6f) [178]. To complete the repair of damaged DNA via HR, eukaryotes can use different mechanisms including BIR (break-induced replication), SDSA (synthesis-dependent strand annealing) or dHJ (double Holliday junctions) [179]. In addition, SSA (single-strand annealing), a non-conservative HR mechanism which results in sequence deletions may be used. SSA depends on homologous repetitive sequences located in proximity to the damage. The model of this HR subpathway assumes that after the resection of 5′ DNA by EXO1, RAD52 promotes the annealing of complementary ssDNA. Non-homologous 3′ overhangs are removed by the nuclease XPF-ERCC1 complex. Finally, the remaining gaps are filled and DNA ends are ligated (reviewed in: [155,178,180,181,182]).

It is believed that the mechanism of nuclear DSBs repair by NHEJ in plant and mammalian cells is similar. The presence of cNHEJ, bNHEJ and MMEJ in plants has been reported [183,184]. An *Arabidopsis rpa1b/atrpa70b* mutant of a human *RPA* homolog showed enhanced sensitivity to UV-B [185]. Acute growth arrest of Arabidopsis with an impaired gene encoding another homolog of the RPA protein, i.e., *rpa1a*/*atrpa70a* was observed following treatment with DSB inducing chemicals [186]. Genes coding cNHEJ proteins including Ku70, Ku80, XRCC4 and LIG4 have been found in *Arabidopsis* and other plants ([187,188], reviewed: [189]). Biochemical studies demonstrated AtKu70/80 complexes with DNA [190]. Whereas plants with defective *AtKu80* [190] and *AtLIG4* [191] gene functions were more sensitive to DSB-inducing agents, the *atpolλ-1* mutant was only mildly sensitive [192]. The up-regulation of *AtKu70*, *AtKu80* [187], *AtLIG4, AtXRCC4* and *AtLIG1* [188] expression in the presence of DSB inducing agents suggests an involvement of the mentioned genes in the repair of these lesions. The role of AtLIG1 in the repair of SSBs and DSBs was confirmed experimentally [193]. An interaction between Arabidopsis AtWEX (a Werner syndrome-like endonuclease) and AtKu70 was demonstrated in vitro implying its role in the repair of DSBs [194]. AtXRCC1, a potential component of bNHEJ in plants, was proved to be involved in the repair of DSBs in an AtKu80-independent manner [195]. Plant homologs of human genes encoding proteins involved in cNHEJ such as DNA-PKcs, XLF, pol *µ*, APLF, PNKP as well as TDT have not been identified yet.

DSBs repair by HR in plants is rare. However, about 30% efficiency of somatic HR between directly repeated sequences located in proximity to DSBs was reported in tobacco [196]. In somatic plant cells almost all DSBs repaired by HR are processed via a SDSA mechanism [197]. Genomic studies have revealed that plants have genes coding for homologs of yeast and human proteins involved in HR [198]. However, so far a functional homolog of human PALB2 has not been found in plants. Studies of Arabidopsis AtMRE11 and AtRAD50 proteins showed that they can form a complex [199]. Moreover, interactions between AtNBS1 and AtMRE11 proteins from Arabidopsis and maize have been established [200]. This suggests that similarly to yeast and human cells, the MRN complex is also formed in plants. Both Arabidopsis *mre11* and *rad50* mutants accumulated chromosomal instabilities associated with DSBs formation [201]. The product of the Arabidopsis *AtRAD52-1* gene, which encodes one of two homologs of the yeast/human RAD52 protein, partially complemented the yeast *rad52* mutant. Moreover, Arabidopsis *rad52-1* and *rad52-2* mutants typically displayed decreased HR frequencies in somatic cells upon treatment with an alkylating agent or mitomycin C [202]. The key role of Arabidopsis *AtBRCA2*, *AtRAD54* and *AtERCC1* genes in somatic HR has also been presented [203,204,205]. Increased frequency of somatic HR and higher sensitivity to DNA damage stress in the Arabidopsis *jsh1* (*jing he sheng 1*) mutant with nonfunctional gene coding for a homolog of human DNA2 protein has been observed [206]. The impact of the Arabidopsis homologs of the human BLM helicase, AtRECQ4A and AtRECQ4B on HR varies. Whereas the *recq4a* mutant was sensitive to DNA-damaging agents and exhibited enhanced frequency of HR in somatic cells, the *recq4b* mutant was not sensitive to these mutagens and displayed strongly reduced HR frequency [207]. The efficiency of somatic HR in Arabidopsis mutants with an impaired *AtXRCC2*, *AtRAD51B, AtRAD51C* and *AtRAD51D* function was markedly reduced relative to that in WT plants [208,209]. *AtRAD51D* and *AtXRCC3*, *Arabidopsis RAD51* paralogues, when transiently overexpressed, increased HR events in *Nicotiana benthamiana* [210]. AtBARD1 (breast cancer associated RING domain 1) deficiency was shown to affect the repair of DSBs by somatic HR under both standard and genotoxic stress conditions [211]. Delayed repair of DSBs was observed in *AtRAD9* and *AtRAD17* plants [212]. This was not surprising since human RAD9 and RAD17 proteins are also known to be engaged in HR [176,213]. Beside the above described proteins attributed to HR based on the homology to human and yeast proteins and experimental verification, also other plant proteins were found to contribute to HR, e.g., AtBRCC36 (lys-63-specific deubiquitinase BRCC36) [214], AtCDKB1 (cyclin-dependent kinase B1-1) and AtCYCB1 (cyclin-B1-1) [215].

Homologs of the prokaryotic proteins related to HR have been identified in plant organelles. This suggests predominant role of recombination-dependent processes in the repair of DSBs in mitochondria and chloroplasts (for a review of DNA repair in plant mitochondria and chloroplasts see: [96]). Based on the functions, plant homologs of bacterial proteins involved in HR can be classified into different groups including DNA protection (AtSSB1—single-stranded binding protein1, AtSSB2), loading of the recombinase (AtODB1—organellar DNA-binding protein1, AtWHY2—Whirly 2, AtOSB1—organellar single-stranded DNA binding protein1, AtOSB2, AtOSB3), formation of the synapsis (AtRECA2—RECA homolog2, AtRECA3), recombination regulation (AtRECX—RECX homolog, AtMSH1), branch migration (AtRADA—RADA-like protein, AtRECG1—ATP-dependent DNA helicase homolog RECG1), DNA synthesis and ligation (AtPOL IB, AtLIG1) [96,216,217]. Involvement of mechanisms similar to MMEJ and NHEJ in repair of DSBs in chloroplasts was also documented [218,219]. In vitro studies verified the role of Arabidopsis organellar DNA pols, AtPOL IA and AtPOL IB, in MMEJ. The results of these studies suggest that the binding of AtWHY2 and AtOSB to ssDNA inhibits MMEJ and favors HR repair of DSBs in plant plastids and mitochondria [184].

## 7. DNA Damage Tolerance

Duplication of nuclear DNA in eukaryotes is dependent on a multi-protein replication complex including pol *α* with the associated primase activity and two replicases, pol *δ* and pol *ε*. A replication complex bound to DNA forms a replication fork whose movement is sensitive to mutations in the template DNA generated by UV light [220]. Unrepaired DNA lesions including pyrimidine dimers may effectively block the synthesis of a new DNA strand throughout stalling of the replication fork [221]. It may be deleterious to a cell eventually leading to the formation of DSBs (reviewed: [222]). To counteract these effects and successfully complete DNA replication, cells may use a DDT (DNA damage tolerance) pathway. DDT may occur by two distinct mechanisms [223,224]. Whereas the first is based on TLS [220], the other is dependent on template switching [225]. DDT studies on a yeast model have indicated that the choice between TLS and the template switching mechanism is controlled by ubiquitination of PCNA in response to encountered DNA damage during DNA replication [223].

**Figure 7:**
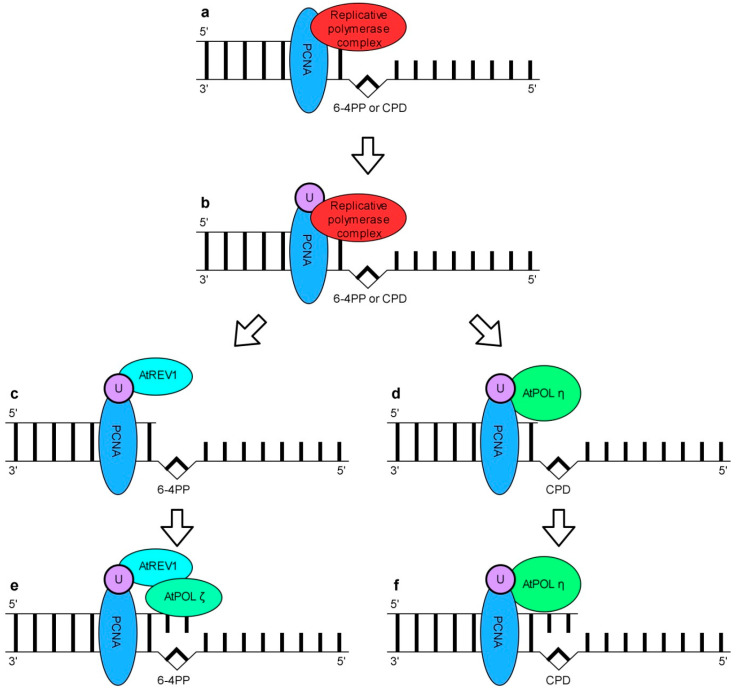
A schematic representation of a translesion synthesis (TLS) model in Arabidopsis. Replicative polymerase complex (either pol *δ* or pol *ε*) are blocked by a CPD or 6-4 PP DNA lesion (**a**). PCNA is monoubiquitynated (**b**). TLS polymerase which has a strong affinity to monoubiquitinated PCNA is recruited and replaces replicative polymerase complex. AtREV1 (**c**) and AtPOL*η* (**d**) are preferentially recruited to CTD and 6-4 PP, respectively. AtREV1 recruits another polymerase such as AtPOL*ζ*, which can add nucleotides regardless the lesion, but is error-prone (**e**). AtPOL*η* synthesis is error free (**f**).

A TLS pathway may either be error-free or error-prone depending on the type of pol involved in DNA synthesis. TLS pols are recruited to the DNA replication machinery at the stalled DNA replication fork site [226]. Replicative pols at the stalled forks may be replaced by TLS pols which incorporate one or more nucleotides opposite the damaged site. Incorrect nucleotide(s) incorporated by low fidelity TLS may be removed either by the exonuclease activity of replicative pols or by MMR. Failure to remove TLS-mediated errors results in the preservation of base substitutions, frameshifts or other types of mutations [227]. In yeasts, PCNA monoubiquitination mediated by RAD6 (ubiquitin-conjugating enzyme E2) and RAD18 (E3 ubiquitin-protein ligase RAD18) proteins enhances the recruitment of TLS pols to a blocked replication fork and regulates the exchange of replicative pols with TLS pols (Figure 7b) [223,224,228,229,230]. Other studies on a human model have revealed that TLS is also regulated by HSP90 (heat shock protein 90) [231].

An error-free DDT subpathway independent of TLS is activated by RAD5 (DNA repair protein RAD5)-mediated polyubiquitination of PCNA [223]. This DDT model postulates filling single-stranded DNA gaps via template switching and recombination involving sister chromatid junctions [225].

Similarly to other eukaryotes, Arabidopsis has several TLS pols including AtREV1, AtPOL*η*, AtPOL*λ*, AtPOL*κ*, AtPOL*θ* and AtPOL*ζ* composed of AtREV3 (DNA polymerase *ζ* catalytic subunit) and AtREV7 (DNA polymerase *ζ* processivity subunit) [220]. Studies of *AtREV1*, *AtPOLη*, *AtPOLζ* gene function deficient mutants have shown the involvement of these gene products in the Arabidopsis response to UV [232,233,234]. AtPOL*λ* has been demonstrated to be responsible for efficient error-free TLS past 8-oxo-dG, a typical DNA lesion induced indirectly by UV-B [235]. AtPOL*κ* was able to extend mispaired primer termini [236]. In vitro, AtPOL*η* effectively bypassed TT CPDs but ineffectively TT 6-4 PPs [234,237]. Recombinant AtREV1 did not incorporate the nucleotides opposite TT CPDs or TT 6-4 PPs but was able to perform in vitro DNA synthesis opposite AP sites. Therefore, it has been proposed that its role is to recruit other TLS-type polymerase(s) to perform the bypass of UV-induced DNA damage [238]. Taken together, these data point to the involvement of TLS in plant response to UV. Given the accumulating information on the eukaryotic TLS pols, the model that describes the bypass of two major UV-induced DNA lesions, i.e., CPDs and 6-4 PPs in plants has been proposed (Figure 7). According to this model TT CPDs are preferentially bypassed by error free AtPOL*η* rather than by other TLS pols such as AtPOL*ζ* which introduces errors into replicated DNA (Figure 7d). In contrary, TT 6-4 PPs bypass requires two TLS pols and cannot be executed by just one. The role of AtREV1 and mutagenic AtPOL*ζ* in this process has been proposed (Figure 7c). Based on the models of a CPDs and 6-4 PPs bypass in plants it was postulated that UV-induced mutations observed in Arabidopsis might be caused by a mutagenic bypass of TT 6-4 PPs by AtREV1 and AtPOL*ζ* [239]. Recent studies indicate that similarly to mammals, AtHSP90 positively regulates the activity of the TLS pathway in *Arabidopsis* [240].

The presence of two alternative DDT pathways in Arabidopsis dependent on either AtREV3 (a subunit of error prone DNA polymerase *ζ*) or AtRAD5a (probably indispensable for the activation of error free DDT in plants) was confirmed by Wang et al. [241]. Given the fact that PCNA has a conserved sequence, structure and functions in the eukaryotes [242,243,244,245], it is highly probable that specific ubiquitination of this protein regulates DDT in plants as well. This hypothesis is supported by the fact that Arabidopsis AtPCNA1 and AtPCNA2 proteins may be monoubiquitinated and polyubiquitinated in a RAD5a-dependent manner [246]. 

## 8. Perspectives and Conclusions

Our knowledge about dark DNA repair mechanisms used by plants to protect their nuclear DNA against deleterious effects of UV is growing. Nevertheless, very little is still known about the mechanisms used to maintain the integrity of genetic material in plastids and mitochondria (for a review of DNA repair in plant mitochondria see: [96]. To conclude, dark repair strategies of DNA lesions are not identical in the nucleus and in the organelles. As mentioned before, the formation of ROS is one of the common effects of UV irradiation. Mitochondria and chloroplasts are ROS sources produced during respiration and photosynthesis, respectively [247]. This renders these organelles especially sensitive to oxidative stress which may result in DNA damage. With the present level of cognition we are still far from detailed understanding of the repair pathways, their interactions and regulation as well as conditions allowing the precise targeting of repair proteins to the nucleus and organelles.

Growing evidence from studies on yeast and animal models indicates that SUMO (small ubiquitin-like modifier), a posttranslational modifier, is an important regulator of the subcellular localization and activity of proteins involved in DNA maintenance including NER, BER, MMR, NHEJ, HR and DDT pathways [248,249,250]. Surprisingly, the data on the role of sumoylation in controlling the stability of plant DNA is scarce. *Arabidopsis* AtMMS21 (methyl methane sulfonate sensitivity 21), a sumo ligase, is a subunit of the SMC5/6 (structural maintenance of chromosome 5/6) complex. Beside the regulation of chromosome dynamics and structure, the SMC5/6 complex is involved in DNA repair. Arabidopsis mutants of SMC5/6 components show moderate hypersensitivity to UV-C [251] (for a review see: [252]). An *Arabidopsis atmms21* mutant displays disturbed somatic HR frequency, indicating that this protein is involved in HR-dependent DNA repair [253]. However, the substrates modified by the AtMMS21 ligase remain unknown [254]. The results of yeast two-hybrid screening followed by the use of the Arabidopsis sumoylation system reconstituted in *E.coli* as well as mass spectroscopy revealed SUMO-conjugates with proteins whose role is attributed to the dark DNA repair in Arabidopsis [254,255,256,257,258] (Table 1).

The sumoylation of tomato and both Arabidopsis PCNA proteins has been confirmed [255,258,260]. In *Saccharomyces cerevisiae* this post-translational PCNA modification regulates TLS and HR of UV-induced DNA lesions [261,262,263]. Another plant protein involved in DNA repair found to be sumoylated is AtRAD4—a homolog of human XPC [264] (Table 1). It has been suggested that in human cells UV-dependent sumoylation of XPC stabilizes this protein [265]. The level of the XPC protein in human cells was shown to determine the effectiveness of pyrimidine dimers repair with a more prominent effect with CPDs [266]. Enhanced UV resistance of *AtRAD4* overexpressing Arabidopsis points to its role also in plant response to UV [267]. The sumoylation of AtCUL4 has been confirmed by Elrouby and Coupland [255]. Along with AtDDB1A and AtDDB2, AtCUL4 forms a complex involved in GGR of UV-induced DNA damage in Arabidopsis [74]. When bound to damaged chromatin, the human DDB2 protein, undergoes sumoylation in a UV-dependent manner [268]. The sumoylation of DDB2 upon UV irradiation was proposed to play, at least in mammals, an important role in the initial recognition and processing of DNA damage induced by UV. Nevertheless, the physiological role of most of the above-described sumoylation events of plant proteins has not been verified. Taken together, it appears that the role of SUMO in maintaining genomic stability in plants in response to UV is still an underestimated area which needs further exploration.

## Figures and Tables

**Table 1:** List of Selected Arabidopsis Proteins whose Role is Attributed to the Dark Repair of UV-Induced DNA Damage whose Sumoylation was Confirmed Experimentally.

Protein	Type of DNA Repair Pathway in Which the Arabidopsis Proteins or Their Human Homologs Is Involved
AtPCNA1 [255,258]/AtPCNA2 [258]	NER, BER, MMR, HR
AtKU80 [257]	NHEJ
AtRAD4 [254]	NER
AtXRCC1 [254]	NER, BER, NHEJ
AtLIG1 [254]	NER, BER, MMR, NHEJ
AtPOL D3 [254]	NER, BER, MMR, HR
AtRCF1 [259]	NER, BER, MMR, HR
AtCUL4 [255]	NER
